# Knowledge, attitude and practices of lifestyle modification and associated factors among hypertensive patients on-treatment follow up at Yekatit 12 General Hospital in the largest city of East Africa: A prospective cross-sectional study

**DOI:** 10.1371/journal.pone.0262780

**Published:** 2022-01-27

**Authors:** Taye Kebede, Zaid Taddese, Abiot Girma

**Affiliations:** 1 Department of Biomedical Sciences and Immunology, Natural Sciences College, Madda Walabu University, Bale-Robe, Ethiopia; 2 ICAP Ethiopia (International Center for AIDS Care and Treatment Programs), The Non-Governmental and Development Organization Home-Based in Ethiopia, Addis Ababa, Ethiopia; 3 Department of Public Health, College of Health Sciences and Medicine, Jimma University, Jimma, Ethiopia; The University of Mississippi Medical Center, UNITED STATES

## Abstract

**Background:**

Hypertension is a devastating global public health challenge; studies indicated that Ethiopia has been affected by the burden of hypertension especially in urban areas. The overall prevalence of hypertension in Ethiopia was estimated to be 19.6% (23.5% in urban and 14.7% in rural population). Along with medical management of hypertension, appropriate lifestyle modification is a crucial and inexpensive means of hypertension control. The main purpose of the study was therefore to assess knowledge, attitude and practice of lifestyle modification among patients on follow up for hypertension treatment at Yekatit 12 General Hospital.

**Methods:**

A prospective cross-sectional study design was applied in Yekatit 12 General Hospital from October 28, 2018, to February 28, 2019, by allotting proportionate samples from the two chronic outpatients departments (OPD). Using single proportion sampling techniques, the study participants were selected and the total sample size calculated was 405. Primarily, clinical measurements were made according to the international standard set to verify true hypertensive patient’s inclusions. Then, data about socio-demographic characteristics, lifestyle modification related to knowledge, attitude and practices were comprehensively collected using an interviewer-administered structured questionnaire. The collected data was entered into Epi-data exported to SPSS Window version of 22 for analysis. All variables with ρ-value less than 0.05 in the final model were considered as independently associated with knowledge, attitude and practices of patients’ lifestyle modification. The strength of association was described by Odds Ratio (OR) at the corresponding CI of 95%.

**Results:**

The overall sampled hypertensive patients as compared to the planned sample size was 95.5% (n = 387), out of which 53.5% (n = 207) was male patients. The mean age was 50 years with a standard deviation of 14.4. The study revealed that 67.7% [95% CI (65.32%, 70.08%)] were knowledgeable; and 54.0% [95% CI (51.34%, 56.6%)] were reported to have favorable attitude towards lifestyle modification. Regarding their practices, 38% [95% CI (19.91%, 57.49%] of the respondents had good practices. Their monthly income [AOR = 2.39, 95% CI (1.12, 5.11)] and duration on-treatment follow up since diagnosed with hypertension [AOR = 4.39, 95% CI (1.20, 16.03)] were independently associated with knowledge. Concerning their damned practices, age [AOR = 7.71, 95% CI (2.4, 24.8)] and knowledge [AOR = 3.94, 95% CI (2.01, 7.72)] were independently associated with the practices.

**Conclusion:**

Though the encouraging high knowledge status and favourable attitudes towards lifestyle modification among hypertensive patients, the practices are among the lowest findings report in all standards. Hence, older patients, jobless patients, and low-income patients and patients on long-term treatment follow up who were diagnosed with hypertension before 10 years needs special attention and interventions by the country NCDs policy formulators to rise their non-pharmacological practices to control high blood pressure and its consequences.

## 1. Introduction

In recent years, Non-Communicable Diseases (NCDs) are also one of the biggest threats to humanity by causing significant mortality and morbidity worldwide including Low-and Middle-Income Countries (LMICs). Among NCDs, Hypertension (HTN), High Blood Pressure (HBP), is common problems [1–3], which affects 1.13 billion people worldwide [2]. Blood pressure (BP) is the force exerted by circulating blood against the walls of the body’s arteries, the major blood vessels in the body. Hypertension is when blood pressure is too high [4], while the level of blood pressure is greater than 140/90mmHg in adults aged 18 years or more [5]. It is commonly termed the silent killer [6]. Hypertension dwindles welfare by causing high mortality and morbidity as well as through its negative health impact in terms of disability, decreased quality of life and mortality associated with stroke and cardiovascular diseases (CVDs) [7–10]. The burden of HTN is felt disproportionately in low- and middle-income countries, where two-thirds of cases are found, largely due to increased risk factors in those populations in recent decades [2]. Unluckily, around 50% of all deaths or disability due to complications of HBP occur among a population with BP <140/90 mmHg which makes the clinical approach to BP only addresses a tiny fraction of hypertension within countries [11, 12].

According to the population-based pooled data to estimate national, regional, and global trends from the 1975 to 2015 report, the global prevalence of HTN in females and males aged 18 years and above was around 20% and 24%, respectively [13]. A decade ago, an estimated 1.39 billion adults aged 20 years or above had hypertension, 694 million men and 694 million women, worldwide. Almost three times as many individuals with hypertension lived in low- and middle-income countries (1.04 billion) than in high-income countries (349 million). In high-income countries, the greatest absolute burden was in the old age groups (60 years and older), while in low- and middle-income countries the greatest absolute burden was in the middle-aged groups (40 to 59 years) [14]. The study from Brazil disclosed a shocking finding where it’s almost a third of the indigenous population had experienced HBP [15]. The top five countries with the highest proportion of men with HBP are found in Central and Eastern Europe [13]. In the first half of the twentieth century, HBP was almost non-existent in African societies. Unfortunately, the top five countries with the highest proportion of women with HBP are all from the African continent; Niger, Chad, Mali, Burkina Faso, and Somalia [16]. Around one in three women in those African countries have been suffering from HBP; implicating the more consequential damage of HTN in women in developing countries than developed nations [17]. A cross-sectional study in Burkina Faso reported a higher prevalence of HBP with increasing age in both rural and urban areas [18]. Like many other African countries, Ethiopia is one of the hardest-hit countries by the double burden diseases [19, 20]. The overall prevalence of HTN in Ethiopia is 19.6% (23.5% in urban and 14.7% in rural areas) [21].

The common dictating factors for the occurrence or exacerbation of HTN are behavioural risk factors such as the consumption of food containing too much salt and fat, eating insufficient fruit and vegetables, harmful level of alcohol intake, lack of exercise and poor stress management [22]. Other factors associated with HTN include socio-economic factors (income, education, and housing), genetics, women with preeclampsia, unplanned rapid urbanization and age [23, 24]. The prevalence of HTN is proportionally raised with obese people, diabetics, people with a family history of HTN and older populations of a given locality [15]. Some studies indicate that the health professionals fail to counsel their clients sufficiently on the importance of the lifestyle management of HTN, while most patients have no information on healthy lifestyle practices [25].

The basic diagnosis and control of HTN are easy. Hypertension is defined as office systolic BP (SBP) values ≥140 mmHg and/or diastolic BP (DBP) values ≥90 mmHg, diagnosed by a validated auscultatory or oscillometric semiautomatic or automatic sphygmomanometers in the OPD offices. On the other hand, one of the common strategies to control HTN is lifestyle modification including healthy diet intake, managing stress, regular exercises, reducing excessive alcohol consumption and abstinence from smoking [22]. In addition, trustworthiness and healthy relationship between health professionals (especially nurses and physicians) and HTN patients have a remission effect on HTN complications [25]. Another risk aversion strategy to prevent the occurrence of HTN and its complications is a motivational interview, which encourages patients to change their unhealthy living habits along with lifestyle modification. Besides, the motivational interview strategy allows the patients to raise their concerns against the planned behaviour and adherence [26].

Lifestyle modification in HTN is also known as non-pharmacological therapy. It is the cornerstone of helping out hypertensive patients to attain lifestyle behaviours that are healthy [27]. The health behaviours and choices are influenced by socio-economic, physical environments, and the commercial environment such as family, social networks, school, workplace, community, and policy. Making a healthy choice is not a simple individual responsibility, nor it is always within his/her power. This causal web of determinants needs a concerted effort of health promoters [28, 29]. As a result, a healthy lifestyle has many diverse elements concerning HTN. For example, a reduction in salt consumption reduces the BP in hypertensive subjects [29]. Generally, having a piece of information about a healthy lifestyle has a significant association with the knowledge of respondents [30]. Moreover, the attitude of clients towards the prevention of hypertension have a bold association with the practice score of respondents [31].

Assessment of the hypertensive patients’ knowledge regarding the acceptable lifestyle practices shows that only 37% of the participants scored above 75%. Among those patients, only 59% have had acceptable knowledge of lifestyle practices concerning their chronic diseases [31]. Similarly, a study reported from Nigeria indicated that 31.7% had good knowledge, 38.3% average knowledge and 30% poor knowledge [32]. Interestingly, the majority of the participants (99%) in the Nigerian study had reported a positive attitude towards lifestyle modification in the management of HTN and the majority of the respondents agreed that HTN is a serious ailment; implicating health-seeking behaviour among the populations covered by the study area [33].

Authors from the United States of America published that around 13% of the African American study participants refuse to shift their dietary behaviour from customary saturated fat to vegetable and fruit-based diet [34]. Good knowledge about salt use, alcohol consumption and smoking effect are essential for successful treatment and steps to be taken to control HTN. In a study applied on 130 participants; 94.6%, 83.8%, and 59.9% had unhealthy knowledge on salt use, alcohol consumption and smoking management, respectively. On top of that, only 39.2% of them knew the importance of a balanced diet. However, their attitude in avoiding salt intake and smoking cigarettes was 94.6% and 98.5% [35]. In Botswana research results, 59.4% took low salt and fat diets, but only 30% of them were used to a normal diet [31]. on the other hand, a study report from Jimma revealed that 5.4% of the participants ate cooked food with salt regularly while a significant majority (94.6%) committed to excluding the addition of salt to their dishes [35].

Regular exercise lowers SPB and DBP by 5 to 10 mmHg [22]. In many studies reported from hospitals, hypertensive patients responded that regular exercise and optimal caloric intake helps to maintain normal body weight [36]. Moreover, in some instances, patients even responded that yoga and meditation are the best practices to resolve stresses and HTN [37]. But, in one Nigerian research report, the majority of the participants fail to differentiate between daily activities and regular exercise [33]. In a cross-sectional study design implemented in Jimma, only 14% claimed their attachment with regular physical activities [35]. In another study elsewhere, integrating higher levels of physical activity among the general population’s lifestyle resolves about 80% of all mortality from cardiovascular disorders. Particularly, performing physical exercise at least three times per week with each exercise sustaining for at least 30 minutes is sufficient to achieve the minimum requirements [36]. Concerning the patients practice towards physical activities; about 67.2% of the respondents responded that they were involved in some forms of physical activity, mostly walking 206 (68.7%) and jogging 100 (33.3%) as reported by the study conducted in one African country [31]. Likewise, 63.6% of the study participants realized the benefits of physical exercise in controlling HBP [8].

Many clinical guidelines of HTN management from some reputable sources indicate that stopping smoking rapidly reduces the risk of HBP [38, 39]. However, there was no difference between smoker and non-smoker participants about the level of knowledge on HTN [31]. Drinking less than 21 units of alcohol a week in men and less than 14 units in women resulted in a reduction of 5/3 mmHg in hypertensive patients [39]. Alcohol is a psychoactive substance with dependence-producing properties and has been widely used in many cultures for centuries. The harmful use of alcohol are diseases, susceptibility to diseases, the social and economic burden in practicing societies [40]. A qualitative cross-sectional survey in Botswana reported 96.4% and 96.6% practices related to prohibiting smoking and reducing the levels of stress, in that order. However, almost equivalent proportions were reported on the knowledge related to restricting themselves from excessive alcohol intake [31].

Hypertension is responsible for at least 45% of deaths worldwide. The current HBP prevalence in some African settings is more than 40% in adults. The prevalence scenario of HTN in Ethiopia is also increasing at an alarming rate [16, 21]. The increasing prevalence of HTN is attributed to population growth and ageing. Also, behavioural risk factors such as unhealthy lifestyles among hypertensive patients in terms of consuming foods containing too much salt and fat, eating less fruit and vegetables, excessive intake of alcohol, lack of exercise and poor stress management [21].

A study report from Ghana indicated that the common complication of HTN in hypertensive patients on follow up treatment is chronic kidney disease (46.9%) [41]. In another study from the adult, Chinese communities on hypertensive patients revealed that cardiovascular diseases such as stroke (33.4%) were the leading cause of death. The Chinese study also emphasized the importance of healthy lifestyle modification as a control mechanism [42]. Interventions implemented on lifestyle modification by a healthcare provider to promote health among hypertensive patients could bring a change. In this randomized controlled clinical trial, the mean score of knowledge, attitude and practice (KAP) of the experimental group right after the study and one month later was significantly higher than that of the control group. This shows the effort of healthcare providers in promoting health education is fruitful in changing the KAP of the hypertensive patients’ lifestyle modification [43].

In research findings addressing the gap of KAP of hypertensive patients towards the lifestyle modification in Jimma, both the knowledge and adherence practice towards lifestyle modification and management of hypertension was poor [35]. In Ethiopia, the few recent burden reports on NCDs in public health institutions are increasing at an alarming rate. In addition, studies addressing the gaps on KAP of hypertensive patients towards lifestyle modification in the country’s main treatment and follow up hub health institutions for NCDs are very scarce. Knowing the gap will contribute to modifying the lifestyle of hypertensive patients in the process of fighting the disease and will give insight into what healthy living looks like. Therefore, this study aimed to assess the KAP of hypertensive patients on treatment follow up towards lifestyle modification and associated factors at Yekatit 12 General Hospital of Ethiopia.

The prevalence measurements of non-communicable diseases like HTN are currently on the rise, though preventable and controllable by easily adopting a healthy lifestyle. In the African context at large and Ethiopia in particular, a few studies have been conducted on the prevention, control and lifestyle modification among hypertensive patients on follow up. Also, the few studies conducted in African countries in different settings showed that there is a significant gap regarding KAP in hypertensive patients towards lifestyle modification to check the advanced progression of HTN and its potential subsequent complications. Those healthy lifestyle modifications are healthy dietary intake (reduction of salt, sodium and fat), feeding habits shift towards more reliance on fruits and vegetables, refrain from smoking, low concentration of alcohol intake, regular physical exercise, maintaining healthy body weight and minimizing stress conditions. Hence, identifying the existing gaps and obtaining scientific shreds of evidence will help the government, non-governmental organizations, and national program planners for putting a pertinent amendment on the existing strategies and developing new policies, which would be executed by the clinicians and other healthcare providers. Above all, the patients and researchers can make use of the findings for future studies to improve the livelihood of the community in a concerted manner. Towards the end, the findings of the current study could stimulate the importance of lifestyle modification in the prevention and control of NCDs with particular reference to HTN. Therefore, this study aimed to assess the knowledge, attitude and practices of hypertensive patients on treatment follow up at Yekatit 12 General Hospital regarding lifestyle modification and associated factors in the highly populated city in eastern Africa, Addis Ababa.

## 2. Methods

### 2.1 Study area and period

Addis Ababa is the first densely populated city in East Africa, the capital city of Ethiopia and subdivided into 10 sub-cities and 99 Kebeles (the smallest unit of dwelling associations). Addis Ababa is the capital of the Africa Union (AU) and the United Nations Economic Commission for Africa (ECA). The city lies between 2200 and 2500 meters above sea level and hosts more than 5.6 million residents. The city has 11 public hospitals, 102 health centres, and 266 pharmacies.

The current study was carried out in the country’s largest chronic diseases referral Hospital, Yekatit 12 General Hospital. Specifically, it was conducted at the two out patients’ department clinics of Yekatit 12 General Hospital. The hospital is one of the eleven public hospitals found in Addis Ababa, is located in the Gulalle sub-city. Relatively, Yekatit 12 General Hospital has fully-fledged and active two chronic outpatient’s department clinics for chronic diseases treatment which deliver services, to all citizens from all corners of the country, from Monday through Friday. The hospital delivers services for at least 1200 hypertensive patients on monthly basis. The daily average number of hypertensive patients getting treatment follow up during the data collection period in both chronic diseases OPD of the hospital was 60 to 80 patients. The actual data collection period in Yekatit 12 General Hospital was from October 28, 2018, to February 28, 2019, after carrying out the pretest on the data collection tools one month ahead (September 2018) in Zewditu Hospital.

### 2.2. Study design

The current study design was an institutional-based cross-sectional study design.

### 2.3 Source and study populations

The source population was all patients attending OPD at Yekatit 12 General hospital for chronic diseases from October 28, 2018, to February 28, 2019. Whereas, the study population was the patients attending chronic OPD for HTN at Yekatit 12 General Hospital. The eligibility (inclusion) criteria were a hypertensive patient on-treatment follow up at least 18 years of age. On the other hand, the exclusion criteria were newly diagnosed (less than 6 months) hypertensive patients at the time of data collection, severely ill HTN patients, and those HTN patients unwilling to give their consent for the current study.

### 2.4 Sample size calculations, sampling technique and sampling procedures

The sample size of this study was calculated using a single population proportion. Determination of the sample size was based on each of the specific objectives of the study, where the ρ-value was adopted from the study conducted in Jimma Zone of Ethiopia in the year 2016 [35]. Benchmarking the Jimma Zone study results in 2016 [35], the final sample size was calculated using single population proportion formula, as follow (Table 1).

$$n{=\frac{1}{d^{2}}(\;Z_{1-\propto /2})}^{2}P(1-P)$$

Where; (**n**) is the minimum sample size, (**P**) is the proportion of hypertensive patients who practice lifestyle modification, (**d**) is the margin of sampling error tolerated, and (**Z**_**1-α/2**_) is the standard normal variable at (1-α) % confidence level.

**Table 1:** The minimum total sample size calculations for the knowledge, attitude and practices of the hypertensive patients’ lifestyle modifications.

Variable	Assumption	Total sample size
Knowledge towards life style modification of HTN patients	P = 0.39(12) Z at 95% (1.96), d = 0.05	n = 365
Attitude towards life style modification of HTN patients	P = 0.94(12) Z at 95% (1.96), d = 0.03	n = 240
Practices towards life style modification of HTN patients	P = 0.41(12) Z at 95% (1.96), d = 0.05	n = 369

The highest calculated sample size by using single population proportion formula was the third specific objective, practices towards lifestyle modification of HTN patients, 369 and by adding 10% non-response rate, the total sample size was 405. This calculated sample size (405), assumes the daily 30 to 40 hypertensive patients’ treatment follow up expected in each of the two chronic OPD clinics of Yekatit 12 General Hospital. Since there are two separates chronic OPD, the average expected number of follow up patients during the data collection period were 5600 (70 patients per day, 1400 patients per month, and 5600 patients over the study period). As all the selected patients have had a patient card for at least 6 months, the patient card number of all the 5,600 patients expected to visit the health facility during the study period, depending on the number of patients estimated in the year preceding our study, 405 hypertensive patients were drawn randomly from all the patients’ list (sample frame) over 4 months, provided they satisfy the inclusion criteria. Hence, by dividing the total number of patients attending the hospital in 4 months (5600) with the planned sample units (405), (5600/405), the sampling class width/interval was 14. To begin with the sampling, the first patient was selected by simple random sampling method (by assigning random numbers using Excel 19 Microsoft office application for Windows) from the list of patients from the 1^st^ to the 14^th^, where the number 8^th^ patient was withdrawn and sampled. Afterwards, every consecutive 14^th^ patient was sampled until the sample size was fulfilled beginning with the 8^th^ patient (i.e., 22^nd^, 36^th^, 50^th^, 64^th^, etc) throughout the study period by systematic random sampling method. The patients were approached in the rooms organized for this particular study by the hospital administrators. Though the total study participants selected were equated to be 405, 18 hypertensive patients were excluded because of inability to fulfil the inclusion criteria of the study, inability to fulfil the international standard of clinical measurements to be hypertensive patient case definitions and a few patients’ rejections to consent for the study.

### 2.5. Study variables

The dependent variable of the study was knowledge of HTN patients towards lifestyle modification in HTN management, the attitude of HTN patients towards lifestyle modification in HTN management, and practices of HTN patients towards lifestyle modification in HTN management. On the other hand, the independent variables were sociodemographic factors (age, sex, educational status, economic status, occupational status, and marital status), health system-related factors (health education), and patient-related medical factors (time elapsed since diagnosed with hypertension and on-treatment follow up for HTN and complications).

### 2.6 Data collection tools and procedures

All participants who accepted to participate in the study were welcomed into the office beside the two NCDs OPD of Yekatit 12 General Hospital. The study is designed to obtain an in-depth interview about the KAP of hypertensive patients towards modifiable lifestyle who were for at least 6 months on antihypertensive drug use, office blood pressure measurement, and anthropometric assessment (weight and height assessment) of the patients.

An interviewer based structured questionnaire was developed to collect data about hypertensive patients KAP towards lifestyle modifications. The questionnaire was translated to Afaan Oromoo, Amharic, and Tigreway local languages spoken commonly in the study area inhabitants and then back-translated to English to check for the consistency and validity of the questionnaire by engaging pertinent linguists from the College of language studies of Addis Ababa University. The questionnaire had six parts, socio-demographic information, questions about KAP of patients’ lifestyle modification, experience and health sector factors questions.

Ethiopia is a multiethnic nation where more than 84 languages are spoken across the country. The capital city of the nation is a diversity hub and home to all these ethnic identities. The largest ethnic group in the country is Oromo. The dwellers surrounding Addis Ababa (the national capital city) are poor Oromo farmers mainly reliant on government hospital health services and are Afaan Oromoo local language speaking who commonly come to the hospital from which we sampled patients. In addition, Amharic, Tigreway, and Gurage local languages are also commonly spoken in the capital city of the country and the patients who get services from the study hospital. To minimize information biases related to language, we (the researchers) hired two nurses for four months who are fluent in at least three of the local languages commonly spoken by the patients utilizing public health institutions, Afaan Oromoo, Amharic, Tigreway, and Gurage local languages for the data collection.

Some in-depth reviews of the available literature regarding KAP standard interrogating questions were made, especially the World Health Organization (WHO) STEPS and The United States Agency for International Development (USAID) developed KAP tools were critically consulted to design the questionnaire for the study. As a result, the interview-based questionnaire was prepared based on the objective of the study (lifestyle modification among hypertensive patients) and the reviewed literature materials. The assigned data collectors (two professional nurses) administered the questionnaire after attending the three days of theoretical and knowledge-based training by the researchers on the objectives of the study, the protocols flow chart, the technique of the data collection, the inclusion and exclusion criteria, privacy, confidentiality and consent issues of the research work plan.

Pretest was applied by the researchers and the data collectors together in another hospital in the city, Zewditu Hospital. Based on the pretest assessment and analysis, a senior epidemiologist was consulted from Jimma University for the possible amendment and modifications on the questionnaire to make it suitable for the respondents and removing jargon. The interview was conducted after each study subject had been informed about the purpose of the study and consented to participate. Alternately, the patients’ clinical parameters; Blood pressure (BP) level, weight, and height were measured and collected from each respondent by the data collectors only through the internationally accepted standard procedures. The whole project scenes were closely monitored and coordinated by the researchers.

#### 2.6.1. Modifiable lifestyle assessment

Smoking status was categorized as current or never/former smoker [44]. A healthy diet was considered as the consumption of at least three portions of fruit and/or vegetables per day [45]. Alcoholism and/or drunk was considered as the excessive consumption of alcohol among patients taking anti-hypertensive drugs as approximated on weekly reference intake concentration [46]. Regular participation in physical activity was considered as involvement in exercise training at least twice a week during the last year. Accordingly, participants were considered physically active or inactive. The active group category patients underwent the activities such as walking for at least 30 min per session, cycling, swimming, running, and resistance exercise [47, 48].

#### 2.6.2. Blood pressure measurement

The office Blood pressure was measured by two well-trained nurses over the whole period of the study in both NCDs OPD of Yekatit 12 General Hospital according to the protocol of Milan EXPO 2015, with a clinically validated Omron M6 electronic sphygmomanometer (Omron, Kyoto, Japan) and in all other samples with a manual sphygmomanometer according to the recommendations from international guidelines [49]. In all participants, the office blood pressure measurements were obtained after resting for 5 minutes in a seated position. An average of three measurements was considered mandatory. Three BP measurements were taken and recorded, with an interval of 1 to 2 min apart between the cuff inflations. Additional BP measurements were taken in cases of the two first readings differ by >10 mmHg, in which case the BP measurement was recorded as the average of the last two BP readings. Manual auscultatory methods were performed in patients with unstable BP values due to arrhythmias, such as in patients with atrial fibrillation, in whom most automated devices have not been validated for BP measurement. A standard bladder cuff (12–13 cm wide and 35 cm long) was used for most patients. Strict care was taken to select the cuff size according to the patient’s arm circumference, where a cuff of arm circumference >32 cm was applied for larger arms [22]. In both OPD of the hospital settings, the assessment was performed in a dedicated office, with an optimal room temperature, and where the privacy of the participants was respected. Blood pressure values were categorized as lower than 140/90 mmHg and equal to or higher than 140/90 mmHg. The duration of antihypertensive drug use was recorded and monitored throughout the sample collection period of the study [1].

#### 2.6.3. Anthropometric measurements

Physical examination provides important indications, such as body habitus. Weight and height measured on a calibrated scale, with the calculation of BMI. Well-trained professional nurses, measured anthropometric indices with participants wearing light, thin clothing and no shoes. Bodyweight was measured through an analogue medical scale. Body height was measured using a standard stadiometer. Bodyweight and height were measured to the nearest 0.1 kg and 0.1 cm, respectively. BMI was defined as the weight (kilograms) divided by the square of height (meters). The BMI cutoffs were applied as per the recommendation of the WHO. The classes of BMI reported by the WHO are 18.5 to 24.9 kg/m2 for normal, 25.0 to 29.9 kg/m2 for overweight, and > 30 kg/m2, for obesity [50, 51].

### 2.7 Data analyses

The collected data were checked for completeness, cleaned and entered into Epi-data version 3.1 and exported to SPSS software. All analyses were performed using SPSS software (Window version 22, SPSS Inc., Chicago, IL, USA). The descriptive data analysis for continuous variables are expressed as mean (SD, standard deviation), and categorical variables as frequencies by absolute value and percentage (%) of the total. Descriptive statistics were used to define demographic and key clinical characteristics of the study population according to the presence of a modifiable lifestyle in hypertensive patients. The descriptive data analysis outputs are presented in the form of tables and graphs. A logistic regression analysis was computed including the four cardiovascular health metrics (smoking, healthy diet, physical activity, and alcohol consumption). Logistic regression analyses, both bivariate and multivariable logistic regression models’ analysis were used to assess the association between the dependent variables and the independent variables, which was between KAP and the modifiable lifestyle in hypertensive patients.

After one on one variable analysis carried out between the dependent and independent variables by the bivariate logistic regression models, variables with a ρ-value less than 0.25 were considered as a candidate for multivariable logistics regression model analysis. Odds Ratio (OR) at 95% confidence interval (CI) was used to measure the strength of the association between the variables. In the final model, a variable with a ρ-value of less than 0.05 was considered as an independent factor associated with KAP modifiable lifestyle (dependent variables) among the hypertension patients on-treatment follow up in the largest and leading chronic diseases treatment Centre and referral hub hospital of the country. To identify some other factors independently associated with the presence/absence of modifiable lifestyle in hypertensive patients, we also estimated the crude odds ratio (COR) and adjusted odds ratio (AOR) at a 95% CI.

### 2.8 Data quality management

To ensure the consistency of data quality, two trained health professionals data collectors were employed for the study period by the reimbursement obtained from one NGO home-based in Addis Ababa city. Every working day (from Monday to Friday) the data collection was monitored by at least one of the researchers to check for the completeness of the questionnaire filled and the measurements of the clinical characteristics. Then after the collected data were entered in Epi-data by the researchers. In this particular study, the official support letter obtained from two government offices, Addis Ababa Administration City Health Bureau and subsequently by Yekatit 12 General Hospital, pave the way for the honest flow of information and data generated. Communicating those letters to each hypertensive patient guaranteed them the full confidence to respond to the questions honestly. Also, it improved the consenting number of the patients significantly after the first day of the data collection, where sharing the copy of the letter (or telling them verbally to those who are unable to read) legitimatized the honest smooth interview interactions between the data collectors and the interviewee.

### 2.9 Operational definitions

**Knowledge** is the fact or condition of knowing or having sufficient information about the lifestyle modification by hypertensive patients.

**Good Knowledge** is answering 50% or more correctly from the 12 standard knowledge questions interrogated to assess the concerning lifestyle modification of hypertension disease.

**Poor knowledge** in answering less than 50% score from the 12 standard knowledge questions administered to the hypertension patients.

**Attitude** is a complex mental status involving beliefs, feelings and values regarding lifestyle modification among hypertension patients. There are five measurement scales for each administered question to assess the attitude of the hypertensive patients towards the lifestyle modifications. **Strongly agree** is when for positive assessment that the participant gave 5 points and 1 point for the negative assessment; **agree** is when for positive assessment the participants gave 4 points and 2 points for negative assessment; **agree to some degree** (certain agree) is when for both positive and negative assessments the participants gave 3 points; **disagree** is when for positive assessment the participant gave 2 points and 4 points for the negative assessment, and **strongly disagree** is when for positive assessment that the participant gave 1 point and allotted 5 points for the negative assessment.

**Favourable attitude is** calculated from the total attitudinal administered questions where each question weight is measured based on the 1 to 5 scales indicated above, where 50% and the above score recorded from the 9 standard attitude assessment questions.

**Non-favorable** is calculated from the total attitudinal administered questions where each question weight is measured based on the 1 to 5 scales indicated above, where less than 50% Score from the 9 standard attitude assessment questions.

**Practice** is to do or perform the component of lifestyle modification among hypertensive patients.

**Good practices** are correctly answering above 50% of 16 standard practices assessment questions.

**Poor practices** are answering less than 50% of 16 standard practices assessment questions.

**Lifestyle modification is** also known as non-pharmacological therapy, which is the cornerstone of helping out hyperactive patients to attain lifestyle behaviours that are healthy including reduction of salt, regular exercise, reduction of alcohol, avoiding smoking, increasing fruit and vegetable and others.

**Reduction of salt** is taking less than 5 grams (2 teaspoons) of salt per day.

**Regular exercise** is performing moderate-intensity exercises for at least 150 minutes a week or 75 minutes of vigorous-intensity exercises a week which would be aerobic physical activity or some equivalent combinations.

**Reduction of alcohol** is about beverages intake limited to 2 drinks per day (20 g/d of alcohol) for men and 1 drink per day (10 g/d of alcohol) for women.

**Smokers** are an active act of smoking a particulate cigarette by the study participant.

**Increasing fruit and vegetable** is taking greater than or equal to 400 grams per day (2–3 serving for each).

**Blood pressure level (BP)** is the pressure of the blood against the inner walls of the blood vessels; where normal BP is BP level of <139/<90 and hypertensive is when the BP level of >139 (SBP) and/or >90 (DBP).

**Body mass index (BMI)** is a way to figure out of healthy weight for height, which is the ratio of weight to height. Underweight is when the BMI is less than 18.5, Normal weight is BMI of 18.5 to 24.9, Overweight is BMI of 25 to 29.9, and Obese is BMI of 30 or more.

### 2.10 Ethical considerations

The ethical approval was obtained from the Institutional Review Board (IRB) of Jimma University. The ethical clearance letter reference number is JU/IRB/00126/18. Also, formal letters of permission were obtained from Addis Ababa Administration City Health Bureau and The Yekatit 12 General Hospital Medical College Dean Office. Each participant was informed about the aims and procedures of the study. Furthermore, written informed consent was obtained from all participants before enrollment. Newly recruited hypertension patients (less than 6 months) and patients below the legal consenting age (below 18 years) were excluded from the study. Besides the purpose of the study, the participants were also informed about the detailed examination of their medical records, and the research potential merits. The data collected was intended for the study purpose. The interview was conducted in a well-aired office near the two NCDs OPD in Yekatit 12 General Hospital, to minimize inconvenience and stress on the patients. Before discharging the patients, pertinent health information messages were delivered to those patients who had had lifestyle malpractices and behaviour, where a persuasive attempt was made in terms of smoking cessation, consumption of less salty foods, significant minimization of alcohol consumptions, and adopting mandatory regular physical exercises.

## 3. Results

### 3.1 Socio-demographic characteristics of the patients

Out of the total participants who attended the treatment follow up unit or clinic of Yekatit 12 General Hospital for chronic ailments during the study period, 405 eligible patients were selected for inclusion into the current study with a response rate of 95.5% (n = 387). The non-response rate was 4.5% (18 patients), which was mainly due to participants refusal to give consent and also the age limit for sampling. Of those 387 sampled patients, 53.5% (n = 207) of the patients were male. The mean age of the respondents was 50 years with a standard deviation of 14.4 years. About 52.7% (n = 204) of the study units were married. Among the total respondents, 65.3% (n = 253) were paid employees and from those employees around 43.1% (n = 129) were self-employed individuals. Of the employed respondents (253), one hundred sixty-six respondents (65.6%) earn a monthly income of 2001 and more Ethiopian Birr (where the current exchange rate of 1 USD is equivalent to 43.58 Ethiopian Birr) during the study period (Table 2).

**Table 2:** Socio-demographic characteristics of hypertensive patients on treatment follow up regarding lifestyle modification at Yekatit 12 General hospital in 2019 (n = 387).

Variable		Frequency	
		Count	%
**Age (in year)**	18–30	40	10.3
	31–45	117	30.2
	46–60	140	36.2
	>60	90	23.3
**Sex**	Male	207	53.5
	Female	180	46.5
**Educational status**	No formal education	58	15.0
	Primary	72	18.6
	Secondary	128	33.1
	College or University	129	33.3
**Marital status**	Single	119	30.7
	Married	204	32.7
	Divorced	38	9.8
	Widowed	26	6.7
Are you currently a paid employee?	Yes	253	65.4
	No	134	34.6
If yes, what is your employment status?	Self-employee	109	43.1
	Govern employee	97	38.3
	Private comp employee	47	18.6
What is your monthly income?	< 1500 Birr	54	21.3
	1501–2000 Birr	33	13.1
	> 2001 Birr	166	65.6

### 3.2 Measurements of clinical conditions of the respondents

During the data collection period, the BP level and BMI of the patients’ clinical parameters were measured. Out of the measured clinical presentations of the respondents, 95.9% (n = 371) of the patients had a BP category record of greater than 139 (systolic reading) and 90 (diastolic reading) (> 139/>90) mmHg. But, the majority of the participants had a normal body weight range (69.2% or 268 patients). Among the total hypertensive patients sampled (387), 40.1% (n = 155) had been on treatment follow up for about 1–5 years in the chronic diseases OPD of Yekatit 12 General Hospital (Table 3).

**Table 3:** Clinical characteristics of hypertensive patients on treatment follow up regarding lifestyle modification at Yekatit 12 General hospital in 2019 (n = 387).

Variable		Frequency	
		Count	%
Body Mass Index	Underweight	20	5.2
	Normal weight	268	69.3
	Overweight	82	21.2
	Obese	17	4.4
Blood Pressure level	Normal (<130/90)	16	4.1
	Hypertensive (> = 130/90)	371	95.9
How long has it been since you have been diagnosed with hypertension?	Half-1 year	112	28.9
	1–5 years	155	40.1
	5–10 years	83	21.4
	> 10 years	37	9.6

### 3.3 Knowledge of hypertensive patients regarding lifestyle modification

Of the total respondents, 67.7% [at 95% CI (65.32%, 70.08%)] was knowledgeable about lifestyle modification. The majority of the respondent patients, 92.0% (n = 356) knew that HTN has another disease consequence and 41% (n = 146) of them mentioned heart disease as the main consequence of HTN. Three hundred thirty-four (93.8%) participants perceived that the consequences of HTN are preventable. A significant number of the sampled patients, 92% (n = 356) knew lifestyle modification could be able to control hypertension. Of those lifestyle modification elements, 90.2% (n = 340) responded that the reduction of salt intake resolves HBP (Table 4). Concerning salt intake reduction, 63.8% (n = 247) of the participants believe that 1 to 2 teaspoons (200g) of salt intake in daily meal curbs HTN, while 92.2% (n = 357) of them raised the importance of using fruit and vegetable in daily meal. Around 95% (n = 371) and 91.7% (n = 355) of the participants responded that alcohol has an effect on HBP and minimizing alcohol intake controls HBP, respectively. Around 79.1% (n = 306) and 73.1% (n = 283) of the participants believed that smoking cigarettes affected HBP and avoiding smoking can control HTN, in that order. A highly significant proportion, 98.4% (n = 381) of the participants thought that performing regular exercise could reduce the level of HBP. A higher proportion of the patients, 97.4% (n = 377) and 96.6% (n = 374) believed that stress could affect HBP and managing stress improves HTN, respectively (Table 4).

**Table 4:** Knowledge of hypertensive patients on treatment follow up regarding lifestyle modification at Yekatit 12 General hospital in 2019 (n = 387).

Variable		Frequency	
		Count	%
Overall knowledge status	Knowledgeable	262	67.7
	Not- knowledgeable	125	32.3
Dose high blood pressure has any consequence?	Yes	356	92.0
	No	31	8.0
If yes to the above, what do you think are the consequences of high blood pressure?	Heart disease	146	41.0
	Renal disease	100	28.1
	Cholesterol	98	27.5
	Diabetes	12	3.4
Can the consequence of high blood pressure be prevented?	Yes	334	93.8
	No	22	6
Do you think modifying lifestyle can prevent hypertension?	Yes	368	95.1
	No	19	4.9
How much salt should be taken per day to prevent high blood pressure?	None	122	31.5
	1–2 teaspoon	247	63.8
	2–4 teaspoon	15	3.9
	4–6 teaspoon	1	0.3
	> 6 teaspoon	2	0.5
How much fruit and vegetable should be taken per day to prevent high blood pressure?	Yes	357	92.2
	No	30	7.8
Does alcohol affect blood pressure?	Yes	372	96.1
	No	15	3.9
Do you think minimizing alcohol intake will help to control high blood pressure?	Yes	335	91.7
	No	32	8.3
Does smoking affect blood pressure?	Yes	306	79.1
	No	81	20.9
Do you think abstinence from smoking will prevent blood pressure?	Yes	283	73.1
	No	104	26.9
Do you believe exercise can help lower your blood pressure?	Yes	381	98.4
	No	6	1.6
Do you believe stresses will higher blood pressure?	Yes	377	97.4
	No	10	2.6
Do you believe managing stress will have prevented high blood pressure?	Yes	374	96.6
	No	13	3.4

### 3.4 Attitude of hypertensive patients regarding lifestyle modification

The highest frequency of the participants, 48.8% (n = 189) strongly agreed that HTN is a preventable disease. Regarding salt intake concentration, around 50.6% (n = 196) strongly agreed and in favour of avoiding extra salt intake in daily meals to control HBP. The majority, 61.2% (n = 237) of the participants strongly agreed that regular exercise helps to control HTN. Among the total participants of the study, 214 (55.3% (n = 214) strongly agreed that dropping cigarette smoking and preventing excessive alcohol intake help to control HTN. Computing from the whole participants of the study, 54.0% [95%CI (51.34%, 56.6%)] had a favourable attitude regarding lifestyle modification for controlling hypertension (Table 5).

**Table 5:** Attitude of hypertensive patients on treatment follow up regarding lifestyle modification at Yekatit 12 General hospital in 2019 (n = 387).

Variable		Frequency	
		Count	%
HTN is a preventable disease	Strongly agree	189	48.8
	Agree	139	35.9
	Some agree	54	14.0
	Disagree	4	1.0
	Strongly disagree	1	0.3
It is good to avoid extra salt intake	Strongly agree	196	50.6
	Agree	154	39.8
	Some agree	33	8.5
	Disagree	3	.08
	Strongly disagree	1	0.3
Good to use extra cooking oil in the daily meal	Strongly agree	1	0.3
	Agree	13	3.0
	Some agree	86	22.3
	Disagree	184	47.5
	Strongly disagree	103	26.6
Good to have whole fruits and vegetables	Strongly agree	206	53.2
	Agree	134	34.6
	Some agree	40	10.3
	Disagree	7	1.8
	Strongly disagree	0	0
BP should be checked regularly	Strongly agree	217	56.1
	Agree	117	30.2
	Some agree	45	11.6
	Disagree	5	1.3
	Strongly disagree	3	0.8
Regular exercise help to prevent HTN	Strongly agree	237	61.2
	Agree	123	31.8
	Some agree	24	6.2
	Disagree	2	0.5
	Strongly disagree	1	0.3
Stopping cigarette and alcoholism help to control HTN	Strongly agree	214	55.3
	Agree	146	37.7
	Some agree	21	5.4
	Disagree	5	1.3
	Strongly disagree	1	0.3
Hypertensive patients should be kept away from stress	Strongly agree	237	61.2
	Agree	123	31.8
	Some agree	24	6.2
	Disagree	2	0.5
	Strongly disagree	1	0.3
Lifestyle modification help to control HTN	Strongly agree	195	50.4
	Agree	153	39.5
	Some agree	32	8.3
	Disagree	6	1.6
	Strongly disagree	1	0.3
Overall attitude status	Favorable	209	54
	Unfavourable	178	46

### 3.5 Practices of hypertensive patients regarding lifestyle modification

Regarding the overall practices, only 38.8% [95% CI (19.9%, 57.49)] of the respondents had good practices concerning preventive and controlling lifestyle modification habits of hypertensive patients. Again, only four in ten hypertensive patients practised the recommended lifestyle modification for controlling hypertension and its potential complications. Regular exercise, stress management, and avoiding stress were the main components practised poorly whereas salt intake reduction, use of fruit and vegetable and moderate alcohol consumption were the components practices well in relative terms (Table 6).

**Table 6:** Practices of hypertensive patients on treatment follow up regarding lifestyle modification at Yekatit 12 General hospital in 2019 (n = 387).

Variable		Frequency	
		Count	%
Lifestyle modification practices to control hypertension			
Salt reduction	Yes	346	89.4
	No	41	10.6
Moderate alcohol consumption	Yes	163	42.1
	No	224	57.9
Regular exercise	Yes	170	43.9
	No	217	56.1
Avoid smoking	Yes	107	27.6
	No	280	72.4
Managing stress	Yes	165	42.6
	No	22	57.4
Minimizing salt concentration from previously adapted amount	Yes	373	96.4
	No	14	3.6
Average amount of salt intake	< 5g (1 teaspoon)	318	84.6
	> 5g (1 teaspoon)	58	15.6
Make use of fruit and vegetable	Yes	333	86.0
	No	54	14.0
Amount of fruit intake	< 200g (2–3 serving)	265	79.3
	> 200g (2–3 serving)	69	20.7
Amount of vegetable intake	< 400g	64	19.2
	> 400g	270	80.8
Ever practiced any alcohol drinking	Yes	304	78.6
	No	83	21.4
Currently habituated to alcohol drinking	Yes	222	73.0
	No	82	27.0
Any attempt to reduce alcohol after being diagnosed with hypertension	Yes	197	87.9
	No	27	12.1
Any practices of cigarette smoking	Yes	123	31.8
	No	264	68.2
Attempt to reduce cigarette smoking	Yes	98	83.1
	No	20	16.9
Still smoking cigarette	Yes	58	15.0
	No	328	85.0
Ever practiced physical exercises	Yes	264	68.2
	No	123	31.8
For how you do physical exercises?	< 15 min/day	121	45.8
	< 30 min/day (< 150 min/week)	115	43.6
	> 30 min/day (> 150 min/week)	28	10.6
Type of physical exercises accustomed to	Walking	67	63.0
	Jogging	68	25.7
	Cycling	27	10.2
Experiebced any major life event previously	Yes	176	45.5
	No	211	54.5
The major life event experienced	Changing residence	73	41.5
	Death of a close family	90	51.1
	Loss of job	13	7.4
Overall practices	Favourable practices	150	38.8
	Unfavourable practices	237	61.2

### 3.6 Health system associated factors role on lifestyle modification of hypertensive patients

**Fig 1:**
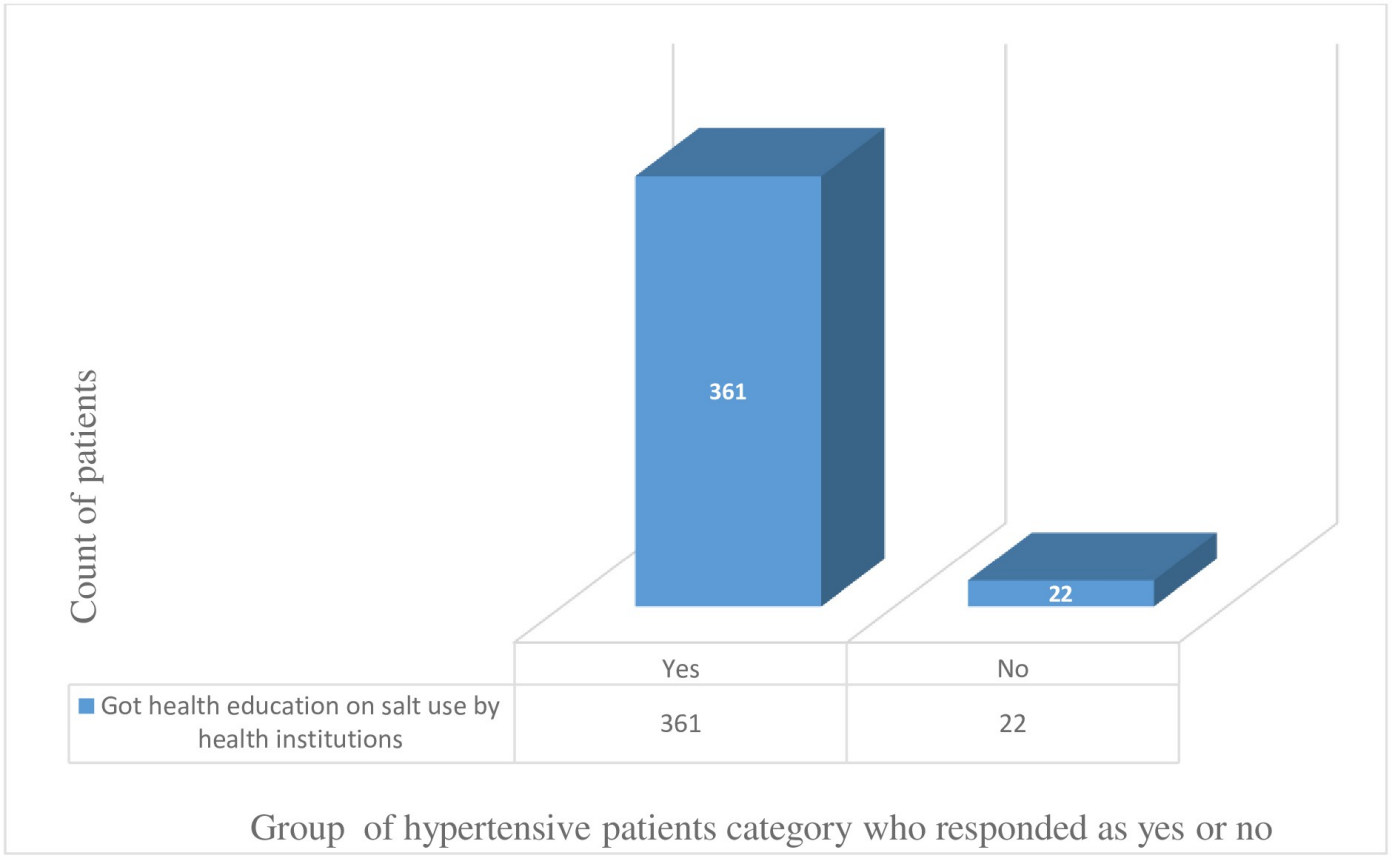
The number of patients who attended health education on salt use in health institutions.

**Fig 2:**
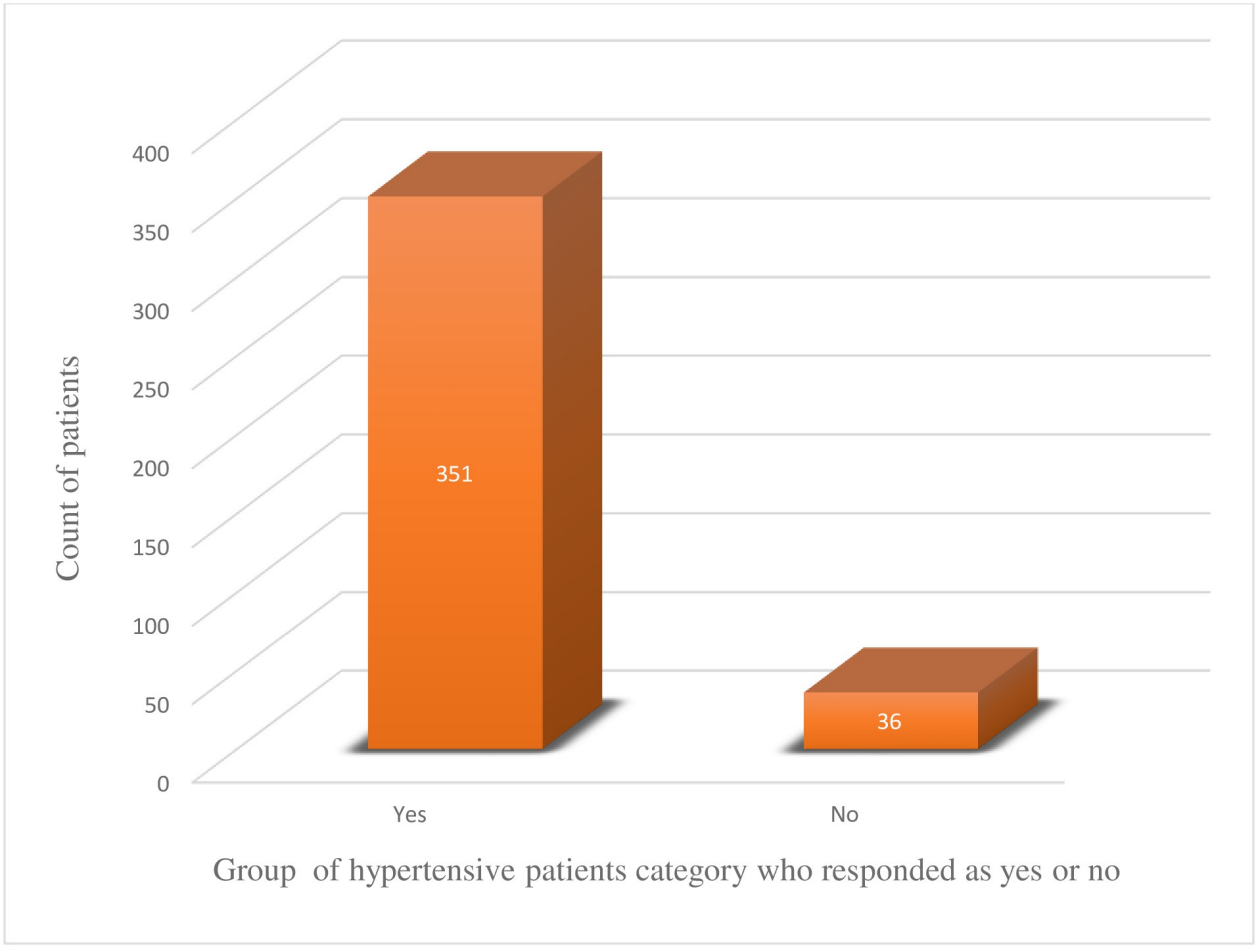
The number of patients who attended health education on regular exercises in health institutions.

The study tried to comprehend additional factors impacting lifestyle modification among hypertensive patients beyond knowledge, attitude and practices. As a result, out of the 387 respondents, a significant majority of the patients acknowledged the health providers for offering health education on the potential danger of too much salt consumptions (361 patients, 94.3%). Also, the health institutions were remunerated respect from the patients’ side for the tireless encouragement to stick the patients to the different regular exercises of their preferences (351 patients, 90.7%) (Figs 1 and 2).

### 3.7 Factors independently associated with patients’ knowledge regarding lifestyle modification

Out of the twelve variables, eight variables (age, educational status, marital status, paid employee, employee status, monthly income, BMI and duration since diagnosed) were identified as candidate variables in bi-variable logistic regression but only monthly income and duration since diagnosed were found to be independently associated (ρ- value,0.05) with knowledge. Those who had a monthly income of 2001.00 or more ETB were 2.39 times more likely to be knowledgeable than those who earn less than 1500.00 or below ETB [AOR = 2.39, 95% CI (1.12, 5.11)]. Those who had been diagnosed with hypertension over the time range of half to one year were 4.39 times more likely knowledgeable than those who were diagnosed with hypertension disease before 10 years [AOR = 4.39, 95% CI (1.20, 16.03)]. Those patients who had been on the treatment follow up for hypertension disease over 1 to 5 years are 6.14 more likely knowledgeable than those participants who had been on hypertension treatment for more than 10 years [AOR = 3.16, 95% CI (1.71, 22.04)] (Table 7).

**Table 7:** Factor independently associated with knowledge of hypertensive patients on treatment follow up regarding lifestyle modification at Yekatit 12 General hospital in 2019 (n = 387).

Variable		Knowledge n (%)		P-value	COR (95%CI)	AOR (95%CI)
		Good	Poor			
Age	18–30	29(7.5)	11(2.8)	0.258	1.60(0.70,3.61)	0.14(0.03,0.70)
	30–45	90(23.3)	27(7.0)	0.022	2.02(1.10,3.71)	0.33(0.09,1.15)
	46–60	87(22.5)	53(13.7)	0.990	0.99(0.57,1.72)	0.31(0.09,0.99)
	> 60	56(14.5)	34(8.9)		1	1
Education	No formal	30(7.8)	28(7.2)		1	1
	Primary	46(11.9)	26(6.7)	0.163	1.65(0.81,3.34)	1.32(0.33,5.33)
	Secondary	86(22.2)	42(10.9)	0.045	1.91(1.01,3.60)	1.63(0.43,6.19)
	College & above	100(25.8)	29(7.5)	0.001	3.21(1.66,6.22)	2.72(0.69,10.7)
Marital status	Single	86(22.2)	33(8.5)	0.012	3.04(1.27,7.25)	0.96(0.44,2.13)
	Married	135(34.9)	69(17.8)	0.050	2.28(1.00,5.20)	2.71(0.61,11.9)
	Divorced	29(7.5)	9(2.3)	0.016	3.75(1.28,11.0)	1.03(0.07,14.0)
	Widowed	12(3.2)	14(3.6)		1	1
Paid employee	Yes	191(49.4)	62(16.0)	0.00	2.73(1.75,4.26)	1.32(0.12,2.33)
	No	71(18.3)	63(16.3)		1	1
Employment	Self	85(33.5)	24(9.5)	0.687	0.83(0.35,1.97)	0.92(0.34,2.51)
	Government	68(26.9)	29(11.5)	0.173	0.55(0.23,1.29)	0.61(0.22,1.67)
	Private company	38(15.0)	9(3.6)		1	1
Income	< 1500	36(14.2)	19(7.5)		1	1
	1501–2000	26(10.3)	8(3.2)	0.275	1.71(0.65,4.51)	2.01(0.68,6.51)
	> 2001	129(50.8)	38(15.0)	0.085	1.79(0.92,3.47)	2.39(1.12,5.11)
BMI	Under weight	12(3.1)	8(2.1)	0.666	1.33(0.36,4.92)	2.13(0.34,13.01)
	Normal wt	193(49.9)	75(19.4)	0.101	2.28(0.44,3.582)	3.24(0.95,11.0)
	Over wt	48(12.4)	34(8.8)	0.671	1.25(0.44,3.58)	1.48(0.39,5.51)
	Obese	9 (2.3)	8(2.1)		1	1
Duration on treatment follow up	Half-1year	81(20.9)	31(8.0)	0.001	3.83(1.76,8.32)	4.34(1.26,14.91)
	1–5 years	115(29.7)	40(10.3)	0.000	4.21(1.99,8.91)	5.74(1.70,19.34)
	5–10 years	51(13.2)	32(8.3)	0.035	2.33(1.06,5.15)	2.65(0.76,9.139)
	> 10 years	15(3.9)	22(5.7)		1	1

### 3.8 Factors independently associated with patients’ attitude regarding lifestyle modification

Out of the twenty-six variables, only five variables (age, marital status, paid employee, employee status and knowledge) were identified as candidate variables in bi-variable logistic regression but none of them has independently associated with the attitude of the hypertensive patients’ lifestyle modifications (Table 8).

**Table 8:** Factors independently associated with the attitude of hypertensive patients on treatment follow up regarding lifestyle modification at Yekatit 12 General hospital in 2019 (n = 387).

Variable		Attitude n (%)		p-value	COR (95%CI)	AOR (95%CI)
		Favourable	Unfavourable			
Age	18–30	20(5.2)	20(5.2)		1	1
	30–45	62(16.0)	55(14.2)	0.774	1.12(0.55,2.13)	0.79(0.32,1.95)
	46–60	91(23.5)	49(12.7)	0.088	1.85(0.91,3.77)	1.12(0.44,2.83)
	>60	36(9.3)	54(13.9)	0.289	0.66(0.31,1.41)	0.79(0.25,2.48)
Marital status	Single	52(13.4)	67(17.4)		1	1
	Married	122(31.5)	82(21.2)	0.005	1.91(1.21,3.03)	1.37(0.78,2.40)
	Divorced	24(6.2)	14(3.6)	0.039	2.20(1.04,4.68)	2.29(0.84,6.19)
	Widowed	11(2.8)	15(3.9)	0.697	0.94(0.40,2.22)	2.11(0.18,24.6)
Paid employee	Yes	147(38.0)	106(27.4)	0.027	1.61(1.05,2.45)	4.32(1.23,10.6)
	No	62(16.0)	72(18.6)		1	1
Employments status	Self	55(21.7)	54(21.3)		1	1
	Government	62(24.5)	35(13.8)	0.052	1.73(0.99,3.04)	1.73(0.99,3.04)
	Private comp	30(11.9)	17(6.8)	0.126	1.73(0.85,3.50)	1.73(0.85,3.50)
Knowledgeable	Yes	150(38.7)	112(28.9)	0.064	1.49(0.97,2.29)	0.97(0.52,1.82)
	No	59(15.3)	66(17.0)		1	1

### 3.9 Factors independently associated with patients’ practices regarding lifestyle modification

Concerning the practices of hypertensive patients’ lifestyle modification; out of thirty-five variables, only nine variables (age, sex, educational status, marital status, paid employee, employee status, time of diagnosis, knowledge and attitude) were identified as candidate variables in bi-variable logistic regression. However, only age and knowledge were found to be independently associated (ρ-value, 0.05) with practices. Those participants aged 18–30 years had 7.71 times good practices [AOR = 7.71, 95%CI (2.4, 24.8)]. Those participants aged 31–45 years were 4 times more likely to have good practices than those aged 60 years or more [AOR = 4.00, 95% CI (1.59,10.03)]. Those who were knowledgeable regarding lifestyle modification were 3.94 times more likely to have good practices than those who were non-knowledgeable [AOR = 3.94, 95% CI (2.01,7.72)] (Table 9).

**Table 9:** Factors independently associated with practices of hypertensive patients on treatment follow up regarding lifestyle modification at Yekatit 12 General hospital in 2019 (n = 387).

Variable		Practices n (%)		p-value	COR (95%CI)	AOR (95%CI)
		Good	Poor			
Age	18–30	25(6.4)	15(3.9)	0.000	6.66(2.92,15.1)	7.71(2.4,24.8)
	30–45	57(14.7)	60(15.5)	0.000	3.80(2.02,7.17)	4.00(1.59,10.03)
	46–60	50(13.0)	90(23.2)	0.012	2.22(1.19,4.13)	3.94(0.8,5.22)
	> 60	18(4.6)	72(18.7)		1	1
Education	No formal	13(3.4)	45(11.6)		1	1
	Primary	19(4.9)	53(13.7)	0.601	1.24(1.32,5.47)	1.57(0.34,7.18)
	Secondary	56(14.5)	72(18.6)	0.006	2.69(1.32,5.47)	3.02(0.72,12.7)
	College & above	62(16.0)	67(17.3)	0.001	3.20(1.57,6.49)	2.40(0.57,10.1)
Marital status	Single	58(15.0)	61(15.8)	0.004	5.23(1.69,16.0)	2.35(5.23,6.23)
	Married	73(18.9)	131(33.8)	0.047	3.65(1.01,9.23)	4.56(1.62,5.23)
	Divorced	15(3.9)	23(6.0)	0.045	3.58(1.02,12.4)	1.23(3.65,5.32)
	Widowed	4(1.0)	22(5.6)		1	1
Paid employee	Yes	119(30.7)	134(34.7)	0.000	2.95(1.84,4.72)	2.23(5.62,8.56)
	No	31(8.0)	103(26.6)		1	1
Employment	Self	49(19.4)	60(23.7)		1	1
	Government	42(16.6)	55(21.7)	0.811	0.93(0.53,1.62)	0.82(0.44,1.55)
	Private comp	28(11.0)	19(7.6)	0.096	1.80(0.90,3.61)	1.41(0.64,3.08)
Sex	Male	86(22.2)	121(31.3)	0.228	1.28(0.85,1.94)	0.88(0.50,1.53)
	Female	64(16.5)	116(30.0)		1	1
Duration on treatment	Half-1year	47(12.1)	65(16.8)	0.030	2.62(1.10,6.24)	1.29(.030,4.67)
	1–5 years	67(17.3)	88(22.7)	0.019	2.76(1.18,6.42)	1.18(0.30,4.67)
	5–10 years	28(7.2)	55(14.3)	0.185	1.84(0.74,4.56)	0.86(0.20,3.59)
	>10 years	8(2.1)	29(7.5)		1	1
Knowledge	Knowledgeable	129(33.3)	133(34.4)	0.000	4.80(2.83,8.14)	3.94(2.01,7.72)
	Not^**ε**^	21(5.4)	104(26.9)		1	1
Attitude	Favourable	92(23.8)	117(30.2)	0.22	1.62(1.07,2.46)	1.31(0.75,2.03)
	Unfavourable	58(15.0)	120(31.0)		1	1

Not^**ε**^ = not-knowledgeable

## 4. Discussion

Hypertension is a chronic condition that leads to serious complications if the person is unable to seek proper control and manage the raised BP level timely. This study demonstrated a significant assessment into the KAP of hypertensive patients who were on follow up a treatment centre in the country’s largest referral hospital for NCDs, Yekatit 12 General Hospital regarding their lifestyle modification and associated factors in the first leading city in Eastern Africa in terms of population density, hosting 5.6 million inhabitants. Primarily, knowledge about lifestyle modification is the key essential part of controlling hypertension; where over two-thirds of the study participants were knowledgeable. Almost half of the study participants had a favourable attitude regarding healthy lifestyle modification, but it is only around one-third of the participants had good practices.

A study conducted in the oil-rich and highly populated African country, Nigeria reported a 60% knowledge score of the hypertensive patients on follow up treatment while assessing the level of KAP of lifestyle modification [33]. These Nigerian paper findings are almost comparable to our study results, which was 67.7% knowledge score (knowledgeable patients). The similarity between the two studies could be because both studies incorporated a similar age group and employed a similar sampling technique (random or lottery sampling technique). The justification for the similarity of the studies may be due to adequate health behaviour interventions in terms of health education focusing on lifestyle modification at health institutions. As a result, patients might have spent a significant amount of time attending health education in healthcare providers and have fewer life concerns at their homes. The knowledge level of the present study is a little bit higher by a factor of 6.7% when compared to this Nigerian study. The possible reason for this minor discrepancy could be because of the larger total sample size in the current study (387 patients), where the Nigerian study sampled a relatively smaller number of hypertensive patients (120 patients).

Among 100 males with hypertension, nearly 84% knew the influence of smoking and alcohol on hypertension and 82% knew at least 3 dietary factors which control hypertension in a study conducted in India [52]. This score is by far larger than the current study findings, which may be because of the small sample size and a single-gender study in the case of India. In another study from (unmentioned study area) Ethiopia, 57% of hypertensive patients had good knowledge of hypertension [53], which is comparable to the present study. The two findings closer score report might be due to the participants’ similarity in awareness levels either through mass media or beneficiaries of healthcare providers education scheme. In another survey study carried out in Zimbabwe, among the sampled 304 individuals who were enrolled in that study, the knowledge score was 64.8% [54], which was reported as poor. However, this Zimbabwean study report is again comparable with the present study, though higher than by a score of about 5%, which could be because of the sample size and majority of study participants from the disadvantaged rural community through the referred system to the hospital.

In this study, it had been seen that those hypertensive patients who earn greater or equal to income 2, 001.00 ETB monthly were found more knowledgeable than those who earned less than 1, 500 ETB per month regarding lifestyle modification. This result is supported by another previous study from Ethiopia, which revealed a highly significant association between patients’ lifestyle modification and knowledge [55]. The similarity might be due to socio-cultural overlap and the availability of health service deliveries. The possible justification about the participants who earn more money could afford medical expenses and go to health institutions to sought follow up treatment and exposure to health education. This implies that whenever an individual earns more money, the need for quality life will be higher and get health education from different sources including the internet, especially in literate subjects.

According to the present finding, patients who had been diagnosed with hypertension in the last 5 years are more likely to be knowledgeable than those who had been on treatment and diagnosed with hypertension before 10 years. However, it is not in line with the previous finding report from Ethiopia five years ago [56]. The disagreement of these two results could be due to the majority of the participants were diagnosed with hypertension in less than 5 years in the current study. The newly diagnosed patients could look for knowledge regarding lifestyle modification to control hypertension, but patients diagnosed before a decade are more likely to be negligent about the control of hypertension disease.

Regarding the attitude, the present study had demonstrated a 54% favourable attitude score. This present finding is lower than the previous study score report from Nigeria, which revealed a skyrocketed and very strong positive attitude (99%) towards lifestyle modification in hypertensive patients [32]. The possible reason for the differences is that the Nigerian study participants had received a health talk forum on lifestyle measurement from health institutions through healthcare service providers [32], unlike the current study. However, both findings suggest a positive attitude towards lifestyle modification in hypertensive patients under the umbrella of health education packages in both countries’ health institutions hosting patients.

Generally speaking, the practice of the study participants towards lifestyle modification in the present study is seriously problematic (38.8%) and not in harmony with many previous research results targeting knowledge and attitude of hypertensive patients’ modifiable lifestyle, where the minority of the participants had good practice score. As an example, in a previous study result report from India, a higher proportion of the rural participants (35.3%) were not practicing dietary modifications such as less daily fruits intake and adding extra salt to their foods [52]. Also, a low score of practice towards lifestyle modifications was reported from rural communities in China [57].

On the contrary, good practices had been documented from the study conducted in eastern Ethiopia [58], wherefrom the total of 200 participants around 50% had a good practice on lifestyle modification recommended for hypertension management. Additionally, a prior study from Ghana concluded that the practices of modifying lifestyle factors (increased physical activity, abstaining from alcohol and smoking, and increased intake of fruits and vegetables) in the study subjects are good [59]. But, an Iranian recent study reported poor adherence and practice to modifiable lifestyle, which is significantly higher in newly diagnosed patients (30.52%) than those who were previously on antihypertensive medication (27.14%) [60]. The variation between those previous findings and the current study might be due to the differences in the socio-economic status of the patients and the availability of the health service providers within the reach of the patients. In our study, the practice of the participants toward lifestyle modification in the control of hypertension is not as expected. This may be because of the poor knowledge and poor adherence to the practices. In addition, healthcare service providers and professionals might not be sufficiently counselling their clients by giving due attention regarding the importance of the lifestyle in the prevention, management and control of hypertension as well as its cost-effectiveness and fewer side effects.

The age of the participants in the current study is one of the independent predictors of lifestyle modifications in hypertensive patients on follow up treatment at Yekatit 12 General Hospital. Those who are in the age range of 18–30 years and 31–45 years were more likely to have good practices towards lifestyle modifications than patients aged 60 years or more. It agrees to the previous work from an African country, Botswana, where poor lifestyle practice is associated with the patient’s older age [31]. These two findings implicate practices of lifestyle modification of patients sampled at hypertension OPD clinic of hospitals is good in younger age, around the age beyond the secondary educational level where more information gathering is intense for prospective life challenges in older age.

In our study, hypertensive patients who were knowledgeable are more likely to have good practices than those who have poor knowledge. By comparing with the previous study conducted in Nigeria, around 64.5% of the current study participants had had sufficient knowledge on salt reduction have had also good practices. On the other hand, the study conducted in Nigeria showed an agreement of good practices in hypertensive patients who had good knowledge regarding regular exercise, excessive alcohol intake minimizations and cessation of cigarette smoking [61]. The possible reason for the mismatch of the two research reports might be because adequate information or good knowledge about lifestyle modification in hypertensive patients scale up the ability to practice.

The clinical implication of the present findings shows that hypertensive patients did not make use of lifestyle modification for the control and prevention of hypertension complications. This warrants the need for more health education interventions at health institutions and community gatherings, as well as individual patient counselling services regarding elements of lifestyle modification. Hence, it is imperative to enhance the knowledge of the hypertensive patients on follow up treatment in OPD clinics of hospitals that could lead to compliance to uninterrupted lifestyle modification in patients seeking treatment behaviour. A closer conclusive remark was reported from a half-decade long study conducted in Ethiopia, where the demographic, personal, social and behavioural factors were associated with lifestyle adherence of hypertensive patients. After controlling possible confounding variable effects on other covariates; the previous study conducted in our country affirmed that the sex, age, patient job status, time since diagnosed with hypertension, co-morbidity, knowledge about the disease, self-efficacy and availability of social support were found significantly associated with the patient’s lifestyle adherence [56]. In this study, around 15% of the hypertensive patients’ behavioural practices were not changed since their treatment commencement. A more or less significantly unchanged proportion of patients’ lifestyle modification practices was verified from a previous cross-sectional study in Ethiopia, which reported 59% of the patients to continue to smoke a cigarette and around 45% of the respondents resisted quitting cigarette smoking [56].

The current study limitations are the inability to include other NCDs threatening the welfare of human beings and only focused on patients aged 18 years and above. Also, it did not sample hypertensive patients from Hospitals with a low record of hypertension patients. Moreover, it did not consider hypertensive patients who did not visit the health institutions during the study period. On the other hand, among the strength of our study, it considers patients from the densely populated city in east Africa, Addis Ababa and sampled hypertensive patients on treatment follow up at NCDs OPD from the robust leading General Hospital (Yekatit 12 General Hospital) found in the Addis Ababa administration city hosting more than 5.6 million residents in particular and the country with 112 million citizens referral destination hospital for the broader rural communities in general. Moreover, strict international standard case definitions of hypertension disease were followed by applying the basic standard clinical characteristics measurements. Furthermore, the fact that we have used a relatively larger sample size, employing computer-generated random sampling methods, minimizing information biases by recruiting multilingual well-trained professional nurses, and using standardized questionnaires to measure the study variables make our study findings robust, valid and reliable. The federal ministry of health of Ethiopia often worries about the number of hypertensive cases and how many of them were put on pharmacologic medications, which ignored the cheaper and yet equally important perspective of hypertension management, the lifestyle modifications. The pieces of evidence generated in the current paper in this regard have paramount importance for planning, and also reflect the performance (commitment) of large referral healthcare facilities on lifestyle medications in hypertensive patients.

## 5. Conclusions

Although health outcomes have improved in low-income and middle-income countries (LMICs) in the past several decades, a new reality is at hand. Changing health needs, growing public expectations, and ambitious new health goals are raising the bar for health institutions in LMICs to produce better health outcomes in terms of hypertension and its chronicles. Staying on the current trajectory is insufficient to meet the population demands. The current study pieces of evidence suggest primary prevention of hypertension and its potential complications in the Ethiopian adult population, especially the wider rural towns communities from all corners of the country referred to the hospital, Yekatit 12 General Hospital. The findings are the test to the Ethiopian repeatedly applauded health extension programs in considering and integrating the non-communicable diseases into its system packages. Lifestyle modification is the mainstay in preventing and controlling hypertension. The present study shows fairly good knowledge and favourable attitude among the hypertensive patients on treatment follow up in Yekatit 12 General Hospital. However, the level of practice is utterly low. Monthly income and duration of treatment follow up are independently associated with the knowledge towards lifestyle modification of hypertensive patients. Also, knowledge and age were independently associated with practices towards lifestyle modification of hypertensive patients. Mitigation measures need to be taken in terms of lifestyle behavioural changes on older patients’ category, unemployed patients, patients receiving lower-income, and patients on treatment follow up for more than 10 years to improve both knowledge and practices of non-pharmacological aspects of patients care as adequate knowledge has a strong association with hypertensive patients’ practices. In addition, patients need to be fully aware of the components of lifestyle modification and its importance in controlling high blood pressure. This study result discloses the need for comprehensive health education and health promotion programs targeting hypertensive patients who are at risk since, as the practices level is marginally below the average. Besides, healthcare staff such as nurses, physicians and other health professionals are advised to provide updated counselling sessions during each patient visit in the hospital outpatient department. Moreover, Hypertensive patients should be better informed about elements of lifestyle modification to complement the deficit and lack of knowledge regarding control of hypertension disease. Furthermore, more importantly, the ministry of health should incorporate the lifestyle modification of hypertension into the packages of grassroots community health workers prevention activities. Amenable policy design and educational support programs for hypertension and its complications should be in place between governmental and non-governmental organizations from the westerners, as exemplified by this study itself. Finally, further studies need to be conducted in the remote regions of Ethiopia (which are not addressed by the current referral system) to determine the bigger picture of knowledge, attitude and practice of hypertension lifestyle measures in the horn of Africa.

## Abbreviations

AU: Africa Union
BMI: Body Mass Index
BP: Blood Pressure
CI: Confidence Interval
CVD: Cardio Vascular Disease
DBP: Diastolic Blood Pressure
ECA: The United Nations Economic Commission for Africa
HBP: High Blood Pressure
HTN: Hypertension
ICAP Ethiopia: International Center for AIDS Care and Treatment Programs in Ethiopia
IRB: Institutional Review Board
JU: Jimma University
KAP: Knowledge, Attitude and Practices
MWU: Madda Walabu University
NCDs: Non-Communicable Diseases
OPD: Out Patients Department
SBP: Systolic Blood Pressure
SPSS: Statistical Packaging for Social Science
UN: United Nations
USAID: The United States Agency for International Development
WHO: World Health Organization
